# Women in the Sick Room

**Published:** 1893-03

**Authors:** 


### WOMEN IN THE SICK ROOM.

There is no place where woman reigns so supreme as in the sick room. There she is a veritable queen, she administers her office and performs the important part of nurse, mother, friend, ofttimes physician, and always benefactor, with a grace peculiarly her own.

A natural born nurse is a blessing, all families should possess at least one. The little mother, we will call her, who early manifests her powers of persuasion and control of baby brother, of sister and of papa himself when he is laid up with rheumatism or malarial fever.

She knows just how to soothe the nerves, to give a foot bath, or a sweat, or a vapor bath, or to make and apply a mustard poultice, or to mix and give the remedy and induce the young child, or feeble grand parent, or crabbed uncle, to take just a little, only enough to cure him.

She can smooth the pillow, change the sheets, give a sponge bath under the bedding, or comb tangled, refractory hair. She can coax the appetite with dainty, favorite dishes, prepared hygienically by her own hands; she can read aloud from favorite authors in a tender, musical, sympathetic voice, till sleep comes softly stealing o'er the languid senses and dreamland enfolds the sick and nervous one in its restful embrace.

This genius of the sick room can bring order out of chaos, can soothe the irritable, comfort the fretful and help restore them to harmony and health.

She it is that brightens the sick room with flowers, and lets in the sun so invitingly that the patient hastens to get well and go out into the world again. The glorious world of sunshine and flowers, of fields, of blossoms and leafy woods and meadows green, where cows, sheep and little lambs are roaming, where the wild flowers stream in rich variety—the daises, the buttercups and the violets blue.

She, it is, who cheers father, comforts mother, soothes little brother or sister, and is so happy and busy that she has no time to be sick or even to think of self.

Her rosy cheeks and bright eyes are an encouragement to others to get well if they are sick and to keep well if not sick.

She loves to read aloud to her patient, and intuitively chooses the right poem, the helpful essay, or the favorite story book. She never tires her listeners, she knows when to stop as well as how to begin. She never asks useless questions that tax her patient, she rests instead of tires her sick charge.

The subtle instinct of the nurse nature divines what to do, anticipates their needs, suggests their real wants, and supplies them quietly and smoothly. A bustler is never a benefit in the sick room, they irritate and retard recovery. A natural born nurse will make a grand success in the duties of the sick room. She will help to cure the patient more positively than the medical prescription. Such a nature is a bouquet of goodness, sweetness, heathfulness and helpfulness. It is a royal inheritance to be a good nurse.

No matter if you are often imposed upon or utilized, at every turn of life's wheel your own soul is expanded and you grow noble by the exercise of your best faculties.

The sick room is a good place to rest oneself, to learn one's powers of usefulness, of preference of value to others. What we do not possess we can cultivate, and by patient persistence, and honest effort rise to the fulfillment of our highest ideal. Try it in the sick room.

